# Grain Boundary‐Driven Lattice Dynamics in a Solid‐State Li‐Ion Conductor

**DOI:** 10.1002/advs.75295

**Published:** 2026-04-17

**Authors:** Jack M. Hemingway, James A. Quirk, Erli Lu, James A. Dawson

**Affiliations:** ^1^ Chemistry ‐ School of Natural and Environmental Sciences Newcastle University, Newcastle Upon Tyne UK; ^2^ School of Chemistry University of Birmingham Birmingham UK

**Keywords:** grain boundaries, lattice dynamics, Li‐ion transport, phonons

## Abstract

Grain boundaries (GBs) are ubiquitous in polycrystalline solid electrolytes and play pertinent roles in determining their ionic conductivity, electronic properties and stability. Despite their importance, the impact of GBs on lattice dynamics in solid electrolytes has not been elucidated. In this work, first‐principles phonon calculations are undertaken on the GBs of a model solid electrolyte, namely, anti‐perovskite Li_3_OCl, to explore their influence on lattice dynamics and the potential consequences for Li‐ion transport. Our results indicate that Li‐ion vibrational hardening exists within the GBs, which suggests an increased barrier for Li‐ion transport, in agreement with both previous experimental and computational studies. Conversely, the anion sublattice in the GBs undergoes vibrational softening, which has been related to an increase in motion and potential for detrimental side reactions, leading to structural degradation and potentially device failure. Furthermore, consideration of the alignment between the Li‐ion migration pathways and eigenvectors of Li‐ion vibrations indicates a poorer alignment on average in the GBs compared to the bulk. This study provides a proof of concept for the simulation of lattice dynamics at solid electrolyte GBs and links the connections made between bulk materials and phonon properties with the phenomenon of GB resistance for the first time.

## Graphical abstract

Grain boundaries in ${\mathrm{Li}}_{3}\mathrm{OCl}$ are shown to significantly alter its lattice dynamics and induce Li‐ion vibrational hardening and anion softening. These changes hinder ion transport while promoting electronic conductivity and instability. Our findings reveal a previously unexplored phonon‐based origin of grain boundary resistance, offering new insight into the design of high‐performance solid electrolytes.

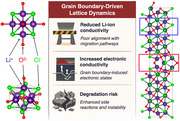

## 1 Introduction

Meeting the energy demands of future generations without a commensurate increase in greenhouse gas emissions is one of the most prominent challenges facing the modern scientific community [1, 2]. Nevertheless, significant improvements in terms of cost, safety and energy density are required to meet the future demands of Li‐ion batteries and it may therefore be necessary to consider alternative battery architectures to achieve these ambitious targets [3, 4, 5].

One such architecture is the solid‐state battery (SSB), which has emerged as an attractive alternative to traditional liquid electrolyte‐based Li‐ion batteries [6, 7, 8]. Replacement of the liquid electrolyte with a solid electrolyte (SE) not only reduces the flammability of the device, thereby improving safety, but can also potentially yield greater energy density and wider operating temperature ranges [9, 10]. Despite these benefits, SSBs also have several downsides that must be addressed prior to their widespread implementation, with one notable example being the growth of metallic dendrites, which propagate from the anode to the cathode through the separator and can cause electrical short circuiting [11, 12, 13, 14, 15]. Given their substantial merits, there has been significant interest in SSBs in recent years, and attempts to relate the structure of SEs to several properties relevant to their application in SSBs have been explored both experimentally [8, 16] and computationally [17, 18, 19].

Atomistic simulations of SEs often focus on the diffusion of migrating ions using molecular dynamics, providing information on migration pathways and the effects of temperature [20, 21]. Such simulations typically require large cells and/or timescales to obtain meaningful statistics and can therefore be computationally expensive, particularly when computed from ab initio methods. Alternatively, there have been numerous reports of correlations between the lattice dynamics (phonon) properties of SEs and their ionic conductivity [22, 23, 24, 25, 26]. Many of these reports suggest that features such as a vibrationally soft lattice, ion‐phonon coupling events and enhancement of the so‐called paddle‐wheel effect (rotation of anion groups enhancing ion transport) could lead to lower migration barriers and therefore increased ionic conductivity in SEs. This is exemplified by the work of Gordiz et al., [27] where targeted excitation of a set of specific phonon vibrations led to a high temperature relative to the remainder of the lattice, resulting in a dramatic increase in the Li‐ion diffusivity of Ge‐doped Li_3_PO_4_. One metric, first reported in the context of ion transport by Muy et al., [23] is the phonon band centre (PBC), which represents the centre of mass of the phonon density of states (g(ω)). Correlations have been reported between the PBC (both total and mobile ion projected) and the migration barrier of mobile ions, suggesting that lower average vibrational frequencies often indicate a decrease in the migration barrier of mobile ions and therefore an increase in ionic conductivity [28, 29]. While these observed trends have been reported for a number of families of similar materials, they tend to break down when large numbers of structurally distinct materials are considered [30]. This is likely due to the in‐built assumption that all phonon vibrations assist in the ion migration, which is typically not the case.

The synthesis of SEs typically involves the sintering and compression of powdered materials, with more modern approaches utilising 3D printing techniques that allow for more precise control over the microstructure [31, 32, 33]. One inevitable result of these processes is the presence of many different nano‐ and polycrystalline regions containing high densities of grain boundaries (GBs). GBs, as surfaces of contact between grains of different orientation, have different local structures and compositions compared to the respective bulk materials. As a result, GBs often present a vast range of ionic conductivities, electronic structures and material properties, which impact the macroscopic performance of not only the SE but also the SSB [34, 35, 36, 37]. It is also known that GBs can contribute to lithium dendrite growth, which can lead to device failure, by acting as pathways for their propagation [37, 38, 39, 40].

Despite the ubiquity of GBs in SEs, computational efforts to explore the ionic conductivity of such materials typically focus primarily on the pristine bulk structures, often resulting in a disconnect between experimental and simulated observations. The inclusion of GBs into an atomistic model of an SE will inherently incur a dramatic increase in computational cost of simulation as GB models are typically much larger and have reduced symmetry relative to the bulk system. As such, examples of computational explorations into the lattice dynamics properties of GBs are scarce and typically focus on simple materials to reduce the computational demand. It is expected that GBs have a softening effect on a materials phonons due to strain, resulting in increased interatomic distances and therefore weaker force constants, as evidenced in simulations of GBs in silicon [41]. This idea of GB phonon softening is at odds with observed GB resistance in SEs, and while there have been several atomistic modelling studies of SEs that have explicitly included GB regions to gain insights into the origin of GB resistance [20, 36, 37, 42, 43, 44], none of these works has explored the influence of lattice dynamics at the GBs.

In this work, we explore the effect of several different GBs on the lattice dynamics of anti‐perovskite Li_3_OCl as a representative solid‐state Li‐ion conductor, using first‐principles simulations. Projected bulk and GB regions are used to qualitatively explore the differences in vibrational characteristics between the different regions and address their influence on the Li‐ion conductivity. We show that Li‐ion vibrational hardening is present at a number of GB structures of Li_3_OCl, suggesting that increased energy is required to excite the ions. The eigenvectors of vibration at the GBs present reduced alignment with the Li–Li migration pathways for three of the four models considered, further suggesting that hopping events are less likely to be vibrationally excited in the GB region when compared to the bulk. This is in agreement with previous reports of significant GB resistance in this material. Moreover, the GBs are found to exhibit anion sublattice softening and reduced bandgaps, which may lead to an increase in electrical conductivity in the device ultimately impacting its efficiency. Overall, this work highlights the need for the consideration of GBs when modeling SEs due to their influence on a wide range of important material properties, such as lattice dynamics, ionic conductivity and electronic structure, all of which can impact the macroscopic function of a SSB.

## 2 Computational Methods

All calculations in this work utilized density functional theory (DFT), as implemented in the Vienna Ab initio Simulation Package (VASP) [45, 46, 47]. More specifically, the PBEsol generalized gradient approximation (GGA) exchange‐correlation functional was employed for each model with a plane wave basis set of maximum energy 600 eV with projector augmented‐wave‐pseudopotentials used in the place of the core electrons [48, 49]. PBEsol was initially selected for bulk Li_3_OCl and subsequently applied to the GB models, motivated by the desire to keep a consistent level of theory throughout this work. Several studies have shown that PBEsol can be applied to GB structures with success [50, 51, 52].

We model GBs in a periodic supercell containing two grains of finite width brought into contact to produce two symmetrically‐equivalent GBs. We ensure that the grains in each model are at least 15 Å thick in order to minimize interactions between the grains. To determine low‐energy GB structures, we performed a systematic scan over all possible rigid‐body translations between the grains, with the translation steps taken to be as close to 0.5 Å as possible whilst allowing the resulting grid of translations to be commensurate with the lattice vectors parallel to the GB plane. The structure with the lowest energy is determined to be a stable structure. This procedure has been used extensively in previous studies to successfully predict GB structures that match well with experiment [53, 54, 55].

Geometry optimization calculations were completed for each structure (bulk Li_3_OCl and four GBs). The bulk was allowed to relax in all lattice directions while the lattice parameters of the GB models were fixed, allowing only ionic relaxation (optimized unit cell parameters for each model are shown in Table S1). The formation energy, *E*_GB_, for each of the four GB models was calculated using Equation (1):

(1) $$E_{\text{GB}}=\frac{E_{\text{total}}-NE_{\text{bulk}}}{2A}$$

where *E*_total_ is the total energy of the GB model, N is the total number of ions in the GB model, *E*_bulk_ is the energy per atom in the pristine bulk and A is the cross‐sectional area of the GB (which for the four models in this work equated to the product of the *a* and *b* lattice vectors for the respective cells, since the GBs are perpendicular to lattice vector *c*).

Γ‐centred grids of k‐points were used for each optimization with the size of the grids outlined in Table S2. The optimized structures of the four GB models were used for the electronic density of states calculations, which once again utilized the PBEsol functional with a much denser 15×15×1 grid of k‐points. Subsequent harmonic phonon calculations were completed using the finite‐difference approach, as implemented in PHONOPY, once again using the optimized structures as inputs [56, 57]. The structures were projected into supercells (dimensions also shown in Table S2), which were sampled using a proportionally smaller k‐point grid than for the optimization calculations. A non‐analytical term correction (NAC) was employed, again using the implementation in PHONOPY, based on the static dielectric constant tensor and Born effective charges calculated from the optimized structures using VASP.

The phonon density of states (g(ω)) was calculated on a sampling mesh commensurate with the supercell for each structure considered in this work, shown in Table S2. The phonon band centre (PBC) was calculated using Equation (2):

(2) $$\mathrm{PBC}=\frac{\int_{0}^{\infty }\omega \;g\left(\omega \right)\;d\omega }{\int_{0}^{\infty }g\left(\omega \right)\;d\omega }$$

where g(ω) can be either the complete phonon density of states or a subset of ion(s) projected phonon density of states. The migration weighted phonon band centre (mPBC) was also calculated using a discrete, mode‐summed version of Equation (2), where the value of the g(ω) is substituted for the alignment of the vibrational mode eigenvector with the Li migration pathway, α. Since the Li^+^ migration in Li_3_OCl is vacancy driven, the migration pathway in this context was simply defined as the neighbouring Li^+^‐Li^+^ vector. As outlined in Equations (3) and (4), for each phonon mode, qν, the value of α was calculated as the absolute maximum of the dot product of the eigenvector of vibration, e, with the available vectors of migration, $\hat{d}$. The average of the number of Li ions considered is taken to yield a single alignment value of the mode.

(3) $$m\mathrm{PBC}=\frac{\sum_{q\nu }\omega_{q\nu }\alpha_{q\nu }}{\sum_{q\nu }\alpha_{q\nu }}$$

where

(4) $$\alpha_{q\nu }=\frac{1}{N_{\mathrm{Li}}}\sum_{i=1}^{N_{\mathrm{Li}}}\max_{j}\left|e_{i,q\nu }\cdot {\hat{d}}_{i,j}\right|$$

**FIGURE 1:**
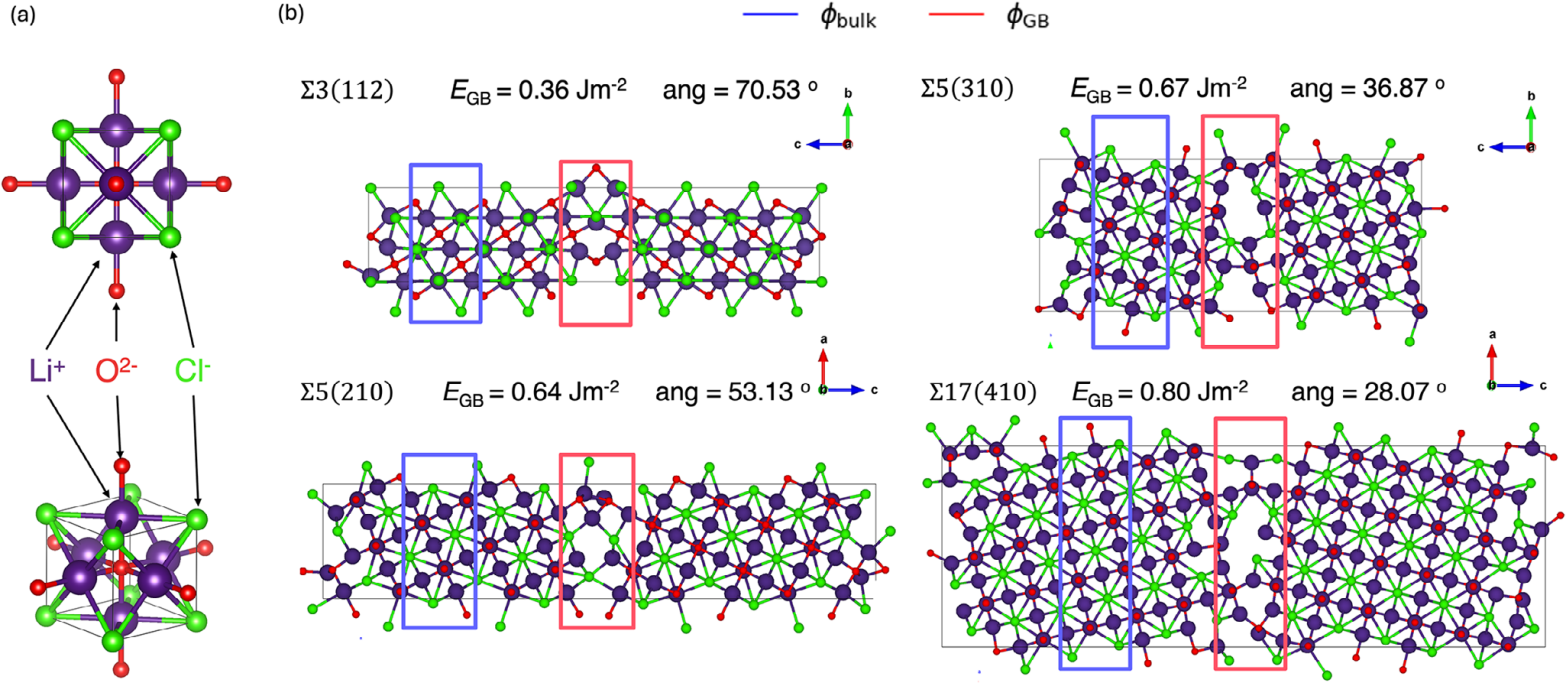
(a) Two orientations of bulk Li_3_OCl unit cell with the ions labeled (Li^+^ ‐ purple, Cl^−^ ‐ green and O^2−^ ‐ red). (b) Optimized structures of GB models with their *E*_GB_ values and misorientation angles outlined alongside the $\phi_{\text{bulk}}$ and $\phi_{\text{GB}}$ regions highlighted in blue and red, respectively.

The projected bulk and GB regions ($\phi_{\text{bulk}}$ and $\phi_{\text{GB}}$, respectively) regions were defined as being ∼5 Å wide, with all ions falling within the range defined being selected as part of the region. This approach has been successfully applied to investigate the different contributions of bulk and GB regions in previous studies [20, 58]. The distance between the bulk and GB regions was maximized to prevent any merging of bulk and GB character. The optimized bulk (unit cell) and GB models of Li_3_OCl are shown in Figure 1. The ions pertaining to the two regions in each GB model are highlighted, i.e., blue and red for the bulk and GB regions, respectively. As a result of the different number of ions in the pristine bulk and $\phi_{\text{bulk}}$ and $\phi_{\text{GB}}$ regions of each of the GB models, the integrals of the g(ω) and projected g(ω) spectra for the bulk and GB regions were normalized to unity prior to direct comparison. The scaled spectra are shown in all plots in this work unless stated otherwise.

## 3 Results and Discussion

### 3.1 Bulk and GB Models

The optimized structure of the pristine bulk is shown in Figure 1a with the respective ion types highlighted. The calculated lattice parameter was 3.839 Å, which is a 1.76 % decrease when compared to the experimental value of 3.907 Å [59]. The GB models were generated to be stoichiometric within periodic cells, with at least 15 Å separating the repeating GB regions to minimize their interaction. Their optimized structures, respective *E*_GB_ values and the $\phi_{\text{bulk}}$ and $\phi_{\text{GB}}$ regions bound by blue and red, respectively, are outlined in Figure 1b. The GB model cells are displayed in the *b*‐*c* plane for the Σ3(112) and Σ5(310) GBs and the *a*‐*c* plane for Σ5(210) and Σ17(410) to highlight the GB that is perpendicular to *c*.

The *E*_GB_ of each of the GB models is calculated to have a low formation energy (in the range of 0.36–0.80 Jm^−2^) relative to other oxide perovskite structures [60, 61, 62] and all exhibit high misorientation angles. The low *E*_GB_ of the various GB structures indicates that their formation is probable and that they are reasonable as representative GBs in Li_3_OCl. Moreover, the Σ3(112) and Σ5(310) GBs are found to be in excellent agreement with previous work (0.36 and 0.64 Jm^−2^ in this work, compared with 0.38 and 0.63 Jm^−2^ in previous work) [37]. It should also be noted that both of these GBs studied in this previous work were found to be stable at elevated temperatures (1200 K).

### 3.2 Influence of Grain Boundaries on Electronic Structure

**FIGURE 2:**
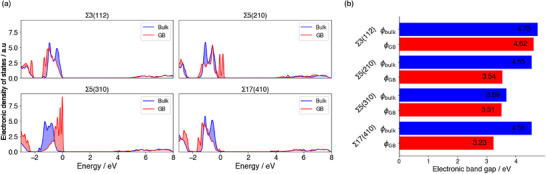
(a) Electronic density of states for each GB model projected onto $\phi_{\text{bulk}}$ and $\phi_{\text{GB}}$ with regions of bulk and GB dominance colored blue and red, respectively. (b) Associated electronic bandgaps for both the $\phi_{\text{bulk}}$ and $\phi_{\text{GB}}$ regions for each GB model.

The influence of the GBs on the electronic structure was explored through calculation of the electronic density of states for the $\phi_{\text{bulk}}$ and $\phi_{\text{GB}}$ regions, with spectra shown in Figure 2a. The energies of the density of states plots are referenced to the valence band maximum (VBM) of the $\phi_{\text{bulk}}$ region, which is set to 0.0 eV. The bandgap energies were also calculated, shown for both $\phi_{\text{bulk}}$ and $\phi_{\text{GB}}$ regions in Figure 2b.

It can be seen from the calculated bandgaps that there is a general decrease in the $\phi_{\text{GB}}$ region compared to the $\phi_{\text{bulk}}$, with a large decrease in the case of the Σ17(410) GB model. There are additional GB localized states present just above the VBM for each of the Σ5(210), Σ5(310) and Σ17(410) GBs, particularly clear in the case of Σ5(210). There is also some increase in the number of occupied states below the conduction band, particularly for the Σ5(210) and Σ17(410) models and is the main cause of the dramatic reduction in bandgap observed for the Σ17(410) GB. Significant narrowing of bandgaps in SEs can promote electrical conductivity as well as suggesting the presence of trapped charges localized in the GB, which have been visualized in previous work [37]. SEs must be electrical insulators to avoid current leakage which would negatively impact the efficiency of the device. It should be noted that the PBEsol functional was used for the calculation of these electronic density of states plots and it is a well known issue of DFT that bandgaps are often underpredicted. While the quantitative value of the electronic bandgaps reported here may be lower than in reality, the qualitative trends are expected to be consistent, as evidenced by previous work using a more accurate hybrid potential (HSE06) on two GBs of Li_3_OCl [37]. Additionally, the use of PBEsol will likely render the visualization of charge density as a broad delocalized state across the GB rather than a localized trapped state as would be achieved with a hybrid functional, as such, these states are not visualized in this work.

In materials where an ion has a number of different charge states (in this example the oxygen ions can exist as either O^2−^ or O^−^) there exists a potential for polaron formation, where a charge carrier induces a local polarization in the lattice causing the charged ion to become trapped [63]. Charge traps or hole formation at the GB could lead to an increased tendency of oxygen ions to change oxidation state therefore potentially giving rise to polarons. Once formed, it is known that polarons can hop between sites, much like a mobile charge carrier. This provides an alternative mechanism for an increase in electrical conductivity in the GB exacerbating potential current leakage [64].

### 3.3 Influence of Grain Boundaries on Lattice Dynamics Properties

**FIGURE 3:**
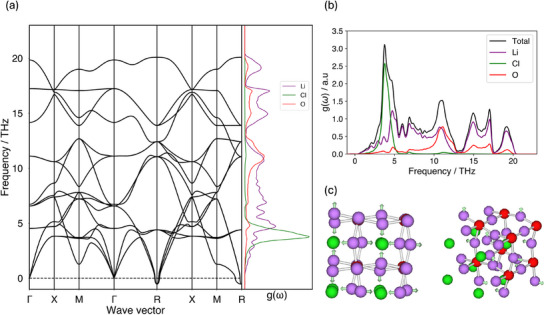
(a) Phonon dispersion spectrum with ion projected g(ω) shown alongside, (b) total and ion projected g(ω) spectra and (c) eigenvectors of unstable phonon at *R* highlighting tilting of the Li_6_O octahedra.

The calculated phonon dispersion and g(ω) from the lattice dynamics calculation of bulk Li_3_OCl are shown in Figure 3. There is instability present at *R* in the phonon dispersion plot, which has been observed previously [65]. Exploration of the eigenvectors of vibration for the unstable mode reveals tilting of the Li_6_O octahedra (eigenvectors of vibration shown from two angles in Figure 3c), suggesting a lower energy phase of Li_3_OCl is accessible. Li_3_OCl has been shown to be thermodynamically unstable and tends to decompose into Li_2_O and LiCl, lending credence to this instability [66, 67]. Moreover, Li_3_OCl is extremely moisture sensitive, with calculations showing an exothermic hydration enthalpy to form Li_2_OHCl [20].

The species projected g(ω) (Figure 3b) shows that the low energy vibrational character is dominated by contributions from Cl ions (green trace), with most of the Cl vibrational character being comprised of one g(ω) peak at ∼4 THz. However, there is some minor Li contribution prior to that at very low frequencies. The contributions from the oxygen ions are scattered throughout the frequency range in small parts, with a major peak just above 10 THz. The Li character also appears to disperse throughout the frequency range, with several pronounced peaks, the largest of which is located just below 5 THz.

**FIGURE 4:**
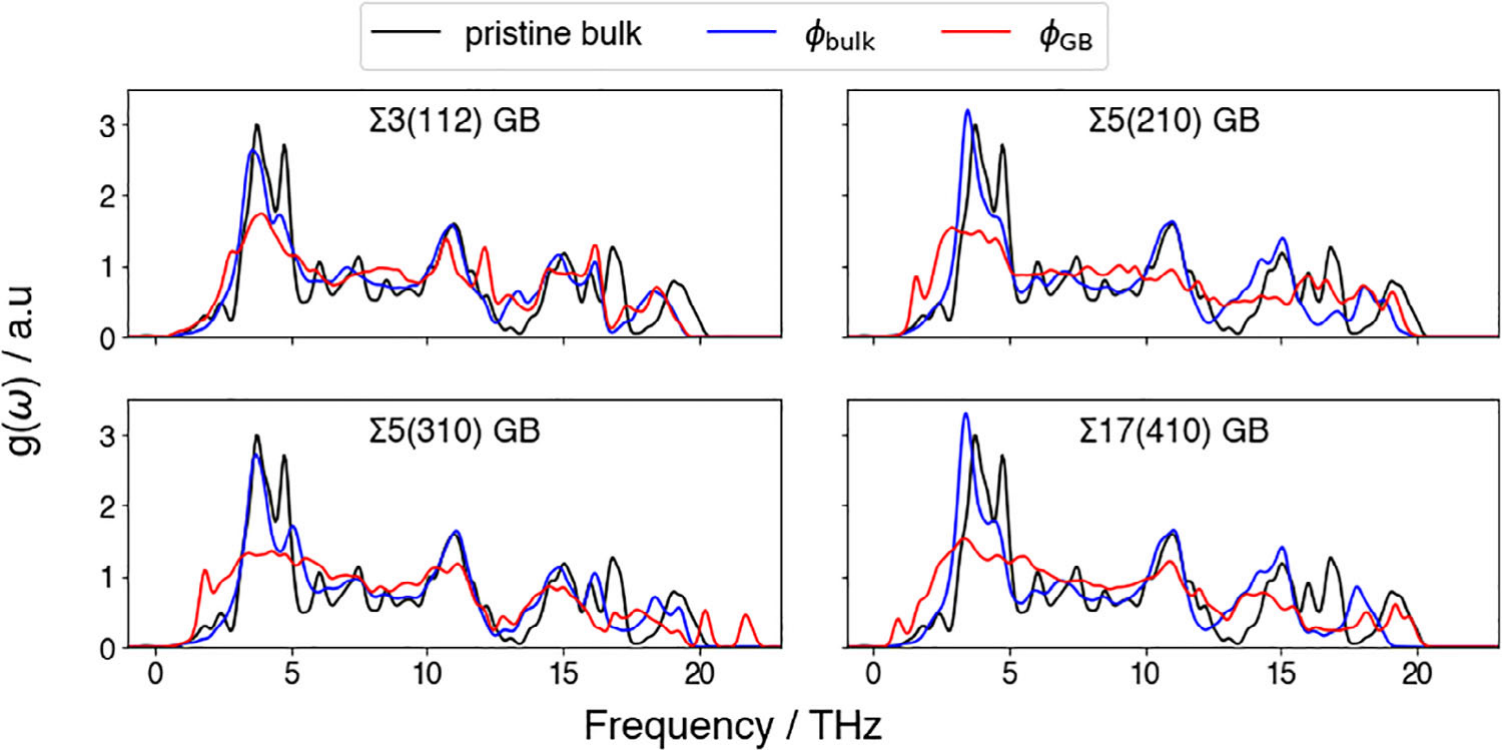
All ion g(ω) trace of the pristine bulk (black) with the all ion g(ω) traces of the $\phi_{\text{bulk}}$ and $\phi_{\text{GB}}$ regions overlaid in blue and red, respectively, for all four GB models considered.

Lattice dynamics calculations were completed for each GB model and the total g(ω) and ion projected g(ω) spectra were obtained. The observed ion projected g(ω) trends observed for the pristine bulk were found to be qualitatively consistent with each of the four GB models (shown graphically in Figure S1). To assess whether the bulk region in the GB models is representative of the pristine bulk, the g(ω) trace of the projected region was compared to that of the pristine system (shown in Figure 4 with pristine bulk in black and $\phi_{\text{bulk}}$ in blue). It is clear from visual inspection that there is generally good qualitative agreement between the g(ω) of the pristine bulk and each of the GB models $\phi_{\text{bulk}}$ regions, particularly at low frequencies (below 10 THz). A general softening of higher energy vibrations is observed for each of the GB models, which may result from influence of the GB region causing small residual strains in the bulk regions. This could potentially be reduced by increasing the GB–GB spacing in the initial models; however, this would incur dramatically increased computational cost due to the increase in system size.

The GB projected spectra are also shown in Figure 4 (red curve) and are markedly different to both the pristine and $\phi_{\text{bulk}}$ traces. This indicates that the vibrational character in the vicinity of the GBs is distinct to that of the bulk region. In particular, there is a pronounced decrease in vibrational character in the feature at ∼5 THz for each of the four models, indicating significant damping of these vibrations. There is also a new low frequency shoulder region to the left of the bulk trace (at ∼3 THz), which is unique to the GBs. This shoulder may form as a result of the expected vibrational softening present at GBs leading to reduced coordination numbers and weaker overall interactions (force constants) between the ions within the GB regions due to the geometric constraints.

**FIGURE 5:**
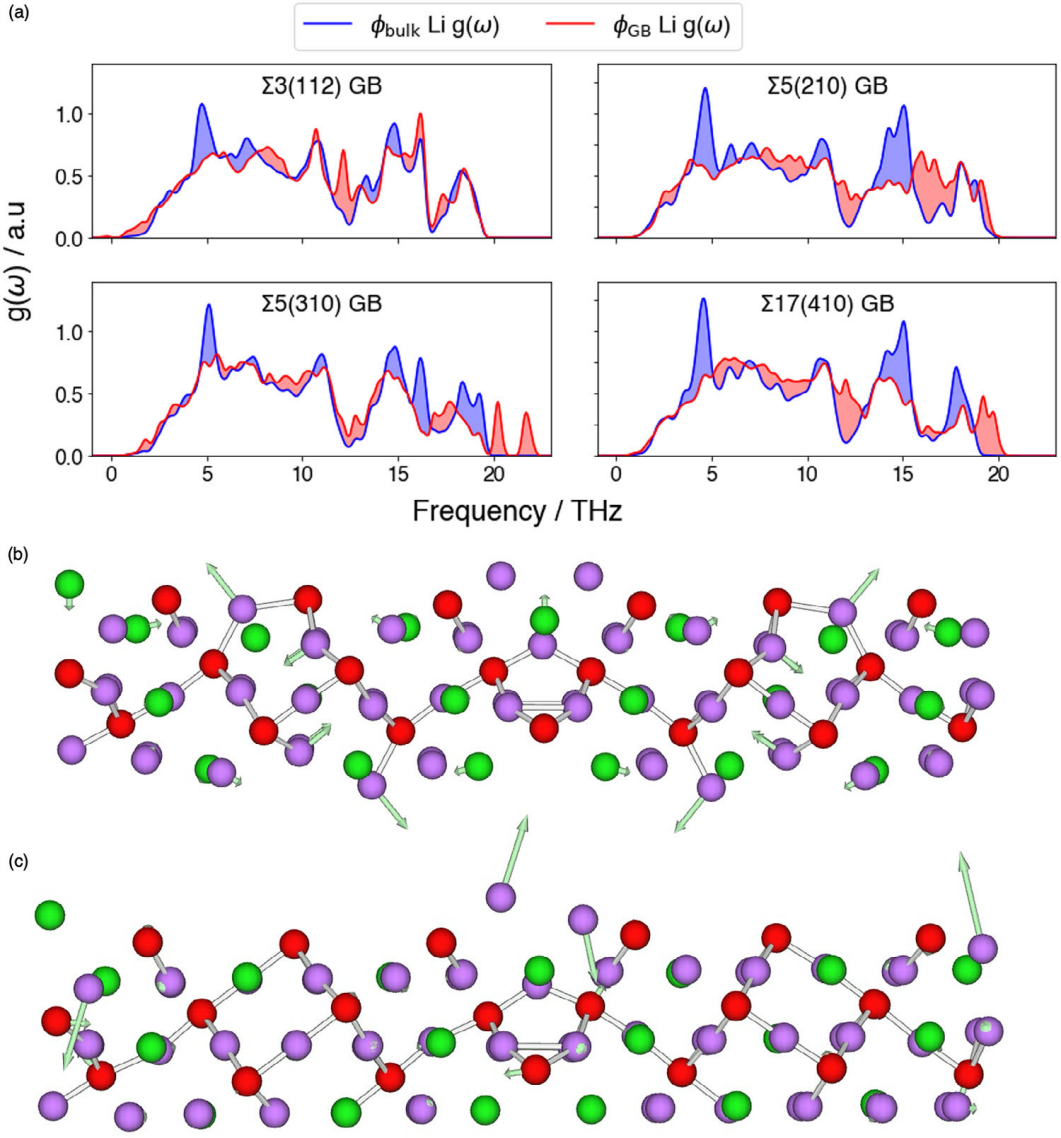
(a) Li‐ion projected g(ω) for each GB model projected onto $\phi_{\text{bulk}}$ and $\phi_{\text{GB}}$ with regions of bulk and GB dominance colored blue and red, respectively. Eigenvectors of vibration of Σ3(112) GB at frequencies of (b) 4.72 and (c) 11.85 THz.

To further examine the effects of the GB region on the motion of Li ions, the ion projected Li g(ω) traces of the $\phi_{\text{bulk}}$ and $\phi_{\text{GB}}$ regions were compared to each other for each GB model (shown in Figure 5a). Frequency ranges over which the $\phi_{\text{bulk}}$ region is vibrationally dominant over the $\phi_{\text{GB}}$ region are highlighted in blue, while the opposite is highlighted in red. Similar trends are present for each of the models considered, with a prominent bulk Li dominance in the peak at ∼5 THz, corresponding to the maximum Li peak in the pristine bulk spectrum. Additionally, each GB model experiences a region of bulk dominance at ∼15 THz in all models. An area of GB dominance is found at ∼12 THz, where in the $\phi_{\text{bulk}}$ and pristine bulk the g(ω) trends toward zero. This increase in character in the vicinity of the grain is most pronounced for the Σ3(112) and Σ17(410) models. The eigenvectors of the phonon modes at 4.72 and 11.85 THz for the Σ3(112) GB (shown in Figure 5b,c, respectively) highlight their localized nature. One interesting feature to note is that the highest energy phonon modes of the Σ5(310) GB appear highly localized to the GB region. This is a feature that has also been reported for a number of tilt GBs of graphene [68]. Previous work has suggested that Li‐ion diffusion at the GB is anisotropic, with diffusion parallel and perpendicular to the GB plane being dramatically different in barrier, with parallel motion favoured [35]. In an attempt to reconcile this with the findings of this work, the ion projected Li g(ω) trace for both the $\phi_{\text{bulk}}$ and $\phi_{\text{GB}}$ regions of each GB model were further split into motions contributing to each coordinate axis (x, y and z ‐ where x and y are parallel to and z is perpendicular to the GB), as shown in Figure S2. While no clear preference for the parallel motion of Li ions can be extracted from the phonon eigenvectors, it is clear that there is increased anisotropy in the $\phi_{\text{GB}}$ region for the Σ3(112), Σ5(210) and Σ5(310) models, which may lead to anisotropic ionic motion.

**FIGURE 6:**
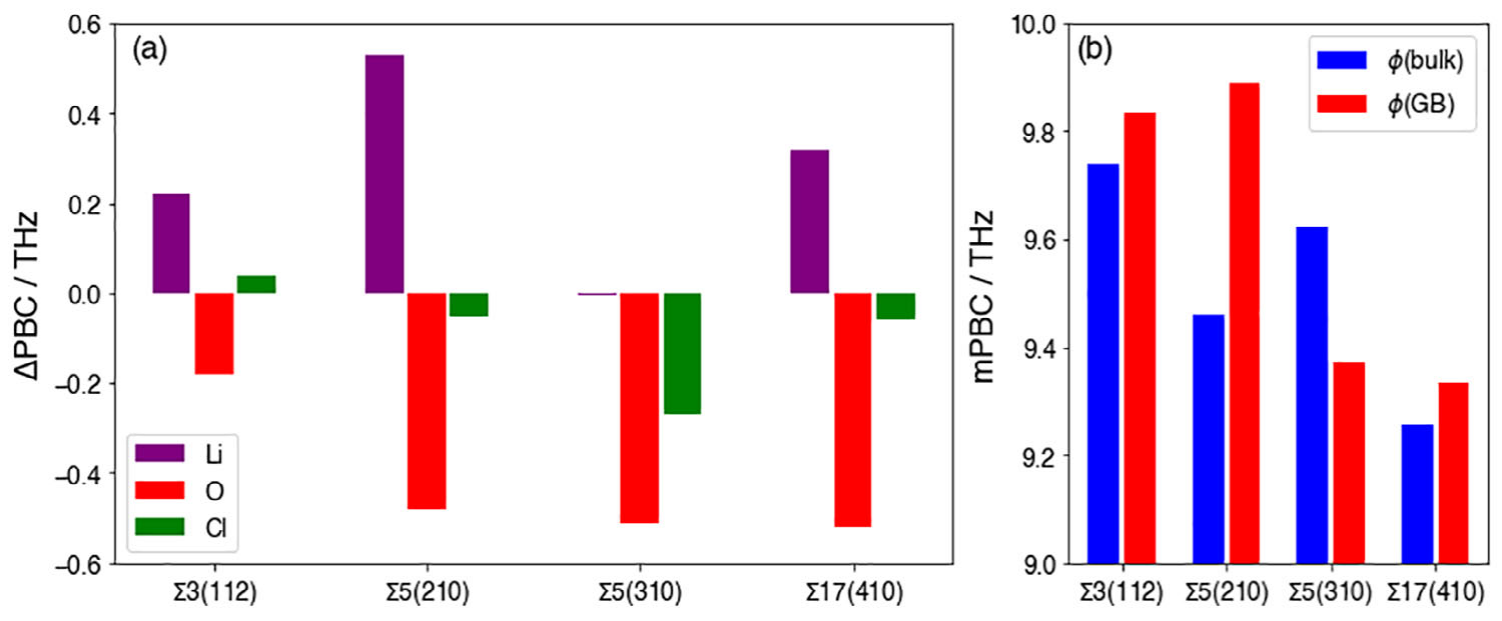
(a) ΔPBC for each ion projection of each GB model with Li, O and Cl shown in purple, red and green, respectively. (b) mPBC for each GB model with $\phi_{\text{bulk}}$ and $\phi_{\text{GB}}$ shown in blue and red, respectively.

The all ion (total) and ion projected PBC values were calculated for both the $\phi_{\text{bulk}}$ and $\phi_{\text{GB}}$ of each GB model, the values for each are shown in Table S3. To explore the difference in PBC between the two projected regions, the change in PBC ($\Delta PBC=PBC_{GB}-PBC_{bulk}$) is shown for each ion projection in Figure 6, where positive values of ΔPBC indicate hardening of the vibrational profile in the GB region and negative values indicate softening.

There are two primary conclusions that can be drawn from the values of ΔPBC, namely, there is an appreciable hardening of the Li vibrations for all GBs with the exception of Σ5(310), where the values of Li PBC for $\phi_{\text{bulk}}$ and $\phi_{\text{GB}}$ regions are identical (10.35 THz). There is also a softening of the anion sublattice (O and Cl ions) for all GB models, with the exception of the Cl vibrations for the Σ3(112) GB, in which there is a small (0.04 THz) increase.

The hardening of the Li‐ion projected PBC values in the GB region is in agreement with previous studies in which appreciable interfacial resistance is observed for the GBs of Li_3_OCl. Previous molecular dynamics simulations on a selection of the Σ3(112), Σ5(210) and Σ5(310) GBs showed an increase in activation energy of Li^+^ migration in the range of 0.11–0.27 eV [35, 37, 69]. Given that these previous studies have clearly established the link between grain boundaries and reduced ion transport in anti‐perovskites, we do not repeat the calculations here. Both the Σ3(112) and Σ5(210) GBs exhibit Li PBC hardening suggesting, on average, an increased amount of energy is required to cause Li motion. Interestingly, there is no observed Li‐ion PBC hardening for the Σ5(310) GB model, despite the previous work suggesting that GB resistance is present for the structure. While the PBC analysis suggests that GB localized Li vibrational hardening is present for three of the four models considered in this work, a drawback of the PBC model as a descriptor for Li conductivity is that it assumes each vibrational mode is of equal importance in assisting migration. This means that modes that strongly align with migration channels will contribute to the PBC an equal amount as modes that do not contribute at all.

In an attempt to mitigate this, the migration‐weighted PBC, mPBC, was defined, where the degree of alignment between the eigenvector of vibration and migration path was calculated for each projected region in each GB model (calculation details given in the Computational Methods). The values of alignment per mode, $\alpha_{q\nu }$, are shown graphically for the $\phi_{\text{bulk}}$ and $\phi_{\text{GB}}$ regions of each GB model in Figure S3. The calculated mPBC values for each region of the GB models are shown in Figure 6b. It is clear that the value of the mPBC of the Σ3(112), Σ5(210) and Σ17(410) GBs is lower in the $\phi_{\text{bulk}}$ region, highlighting vibrational hardening of the Li sublattice in the GBs. This suggests that the migration of Li along migration pathways is more favourably excited at lower frequencies in the bulk region than the GB, aligning with the reported GB resistance. It should be noted that the opposite trend is once again found for the Σ5(310) GB model, suggesting that the GB region actually improves the Li‐ion motion along the migration channels. While previous work found that the Σ5(310) GB was less resistive than the Σ3(112) GB, it nevertheless increased the Li‐ion migration activation energy [37]. This highlights that other mechanisms outside of phonon assisted ion hopping are important to consider, such as changes to the electrostatic potential, when assessing the influence of GBs on ionic migration. Moreover, it has been reported that the redistribution of Li ions and vacancies in the Σ3(112) GB of Li_3_OCl can result in an increase in their migration barrier [70]. The inclusion of such vacancies to reconcile this change with lattice dynamics properties will be an important consideration in future applications of this approach. One further thing to consider is whether the separation between the two GB regions in the cell of the Σ5(310) GB (∼15 Å‐ the smallest considered in this work) is large enough. This separation could lead to residual strain in the $\phi_{\text{bulk}}$ region which will alter the structure during the geometry optimization process.

Softening of the anion sublattice has previously been shown to reduce the oxidation potential of a series of LISICON SEs [23]. It has been suggested that the downward shift in the vibrations of the anion sublattice leads to increased anion motion, thereby lowering the kinetic barrier to a number of decomposition reactions. The observed softening of the anion sublattice at the GBs in this study may therefore have a negative influence on the stability of the SE (and SSB) through the promotion of its degradation through these side reactions. This finding is also in agreement with the observed bandgap narrowing of the four GB models considered in this work, as the bandgap can be used as a lower estimate of a SE's electrochemical stability window [71]. Reactions localized to the GB may also influence the formation of polarons at the GB, the migration of which will likely also be influenced by the change in local structure and lattice dynamics at the GB. It has also been suggested that Li_3_OCl when utilized as a glass not only exhibits Li^+^ conductivity, but also Cl^−^ conductivity. As such, softening of the anionic sublattice may lead to increase in Cl^−^ transport, followed by build‐up and subsequent reaction of Cl^−^ at the electrode, leading to eventual device failure [72, 73]. Finally, an increased propensity for destructive reactions at the GB could also suggest that the GBs in Li_3_OCl are somewhat responsible for the sensitivity to water, and that hydration reactions may be localized to the GB.

## 4 Conclusions

Understanding the effects of GBs on the ionic migration of polycrystalline solid electrolytes is vital for the development of high‐performance SSBs. Unfortunately, this has been limited by the numerous experimental and computational challenges associated with these interfaces. Here, we have explored the influence of different GBs on the lattice dynamics of a representative solid‐state Li‐ion conductor for the first time. Our results reveal the following key features:

1. There is a general hardening of the average vibrational frequency of the Li‐ion projected motions in the $\phi_{\text{GB}}$ region relative to the $\phi_{\text{bulk}}$. This suggests an increase in energy is required to excite these vibrations, meaning that phonon‐assisted Li‐ion hopping will be less likely in the GB region, thereby corroborating the observed GB resistance in this material.
2. There is a general hardening of vibrational modes that are aligned with the Li‐ion migration pathway for the Σ3(112), Σ5(210) and Σ17(410) GB models. This suggests that the modes that are likely to assist Li‐ion migration are damped at the GBs. This finding represents a new mechanism through which reduced ion transport at GBs in SEs can be understood.
3. There is a decrease in bandgap in the $\phi_{\text{GB}}$ region relative to the $\phi_{\text{bulk}}$ and softening of the anion sublattice at the GBs. Both of these effects can potentially lead to reduced SE stability and greater susceptibility to dendrite formation.

In addition to the fundamental understanding of how GBs inhibit ion transport, this work acts as an important proof of concept for the exploration of the lattice dynamics of polycrystalline SEs that can be built on in further studies. For example, the rapid development of machine learning interatomic potentials will enable us to explore larger models with greater separation between the bulk and GB regions to rule out residual strain effects and study an increased number of GB structures. Furthermore, the inclusion of potentially influential anharmonic contributions to the lattice dynamics could also be captured using molecular dynamics simulations. Once again, due to the inherent cost of these calculations, they will likely require machine learning interatomic potentials to be computationally viable. The use of molecular dynamics simulations would also allow a direct measure of the ionic conductivity of such models, which could be used in conjunction with the lattice dynamics properties. Finally, we expect the methods and findings described here to be applied to a range of other SE families to confirm whether the trends observed in this work are applicable to all GBs or are instead material dependent.

## Conflicts of Interest

The authors declare no conflicts of interest.

## Supporting information


**Supporting File**: advs75295‐sup‐0001‐SuppMat.pdf.

## Data Availability

The data that support the findings of this study are available from the corresponding author upon reasonable request.
