# Supplementary material for: Grain Boundary‐Driven Lattice Dynamics in a Solid‐State Li‐Ion Conductor
Source: Adv Sci (Weinh). 2026 Apr 17;13(38):e75295. doi: 10.1002/advs.75295 (PMC13335075; doi:10.1002/advs.75295)
Supplement: Supplementary file 1 — Supporting File: advs75295‐sup‐0001‐SuppMat.pdf. [file ADVS-13-e75295-s001.pdf]

——— Supporting Information ———

Grain Boundary-Driven Lattice Dynamics in a Solid-State Li-Ion Conductor

Jack M. Hemingway,*,† James A. Quirk,† Erli Lu,‡ and James A. Dawson*,†

†*Chemistry - School of Natural and Environmental Sciences, Newcastle University Newcastle Upon Tyne, NE1 7RU, UK*

‡*School of Chemistry, University of Birmingham, Birmingham, B15 2TT, UK*

E-mail: jack.hemingway@newcastle.ac.uk; james.dawson@newcastle.ac.uk

Table S1: Unit cell dimensions of the structures considered in this work.

**Structure**	**a / Å**	**b / Å**	**c / Å**	α / °	β / °	γ / °
Bulk Li_3OCl	3.839	3.839	3.839	90.0	90.0	90.0
Σ3(112) GB	5.462	6.690	32.168	90.0	90.0	90.0
Σ5(210) GB	8.757	3.916	42.284	90.0	90.0	90.0
Σ5(310) GB	3.863	12.214	30.707	90.0	90.0	90.0
Σ17(410)	16.148	3.916	48.544	90.0	90.0	90.0

Table S2: k-point grids used for both the optimisation and phonon calculations, supercell dimensions used in the phonon calculations for the bulk structure of Li_3OCl and the four GB models as well as the g(ω) sampling meshes.

**Structure**	**Optimisation k-points**	**Phonon supercell**	**Phonon k-points**	**g(ω) sampling mesh**
Bulk Li_3OCl	6x6x6	6x6x6	1x1x1	18x18x18
Σ3(112) GB	3x3x1	3x3x1	1x1x1	9x9x1
Σ5(210) GB	2x4x1	2x4x1	1x1x1	6x12x1
Σ5(310) GB	4x2x1	4x2x1	1x1x1	12x6x1
Σ17(410) GB	1x4x1	1x4x1	1x1x1	3x12x1

Table S3: Calculated values for the total (all ion), Li, O, and Cl PBCs. All values are given in THz.

**GB models**	**Total**	**Li**	**O**	**Cl**
Σ3(112) ϕ_{bulk}	9.06	10.12	10.96	4.00
Σ3(112) ϕ_{GB}	9.30	10.34	10.78	4.04
Σ5(210) ϕ_{bulk}	8.93	9.96	10.98	3.84
Σ5(210) ϕ_{GB}	9.05	10.49	10.50	3.79
Σ5(310) ϕ_{bulk}	9.26	10.35	11.11	4.13
Σ5(310) ϕ_{GB}	9.10	10.35	10.60	3.86
Σ17(410) ϕ_{bulk}	8.82	9.85	10.78	3.76
Σ17(410) ϕ_{GB}	8.64	10.17	10.26	3.70

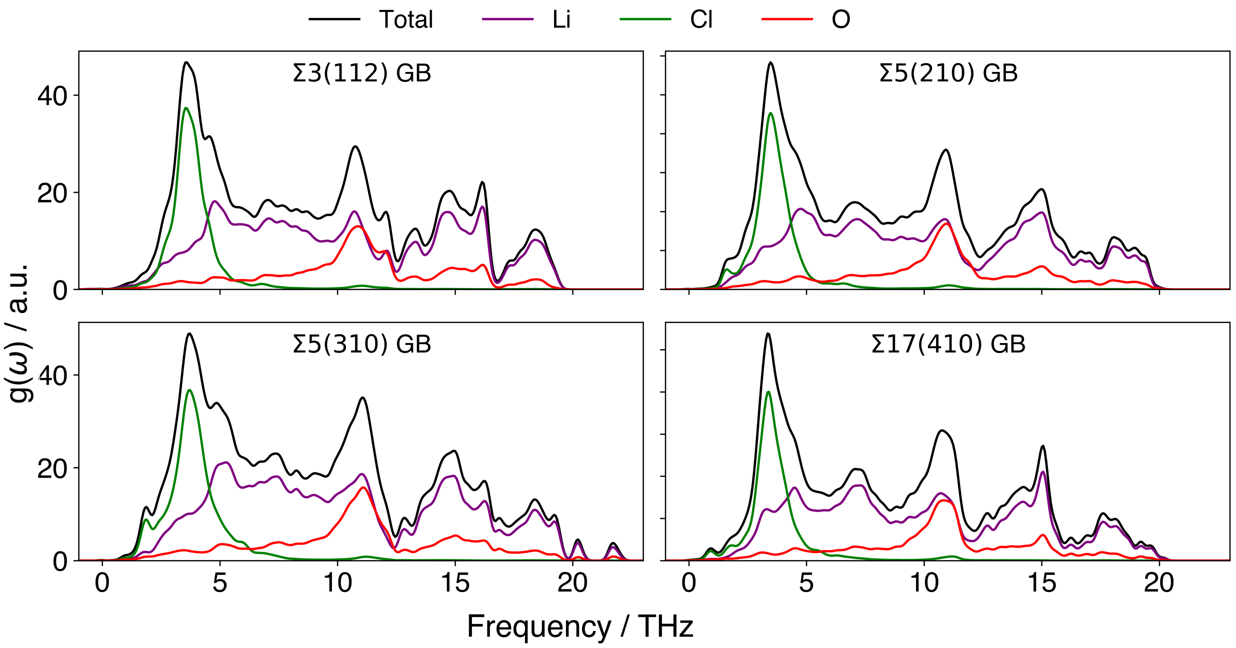

Figure S1: Total and ion projected g(ω) traces for the complete cells of each of the four GB models considered in this work.

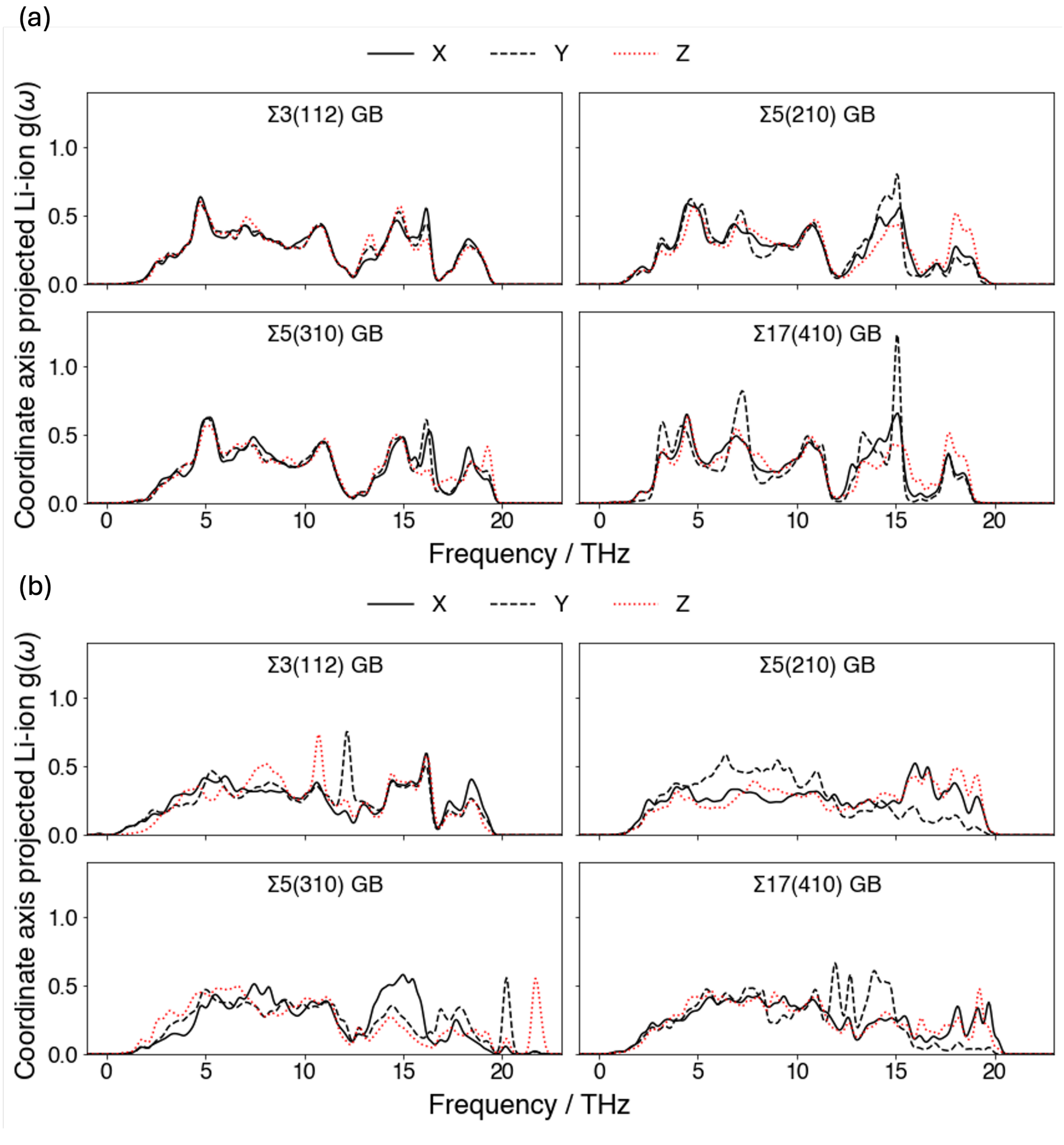

Figure S2: Coordinate axis separated Li-ion g(ω) traces for the ϕ_{bulk} region (a) and ϕ_{GB} region (b) with the z axis motion (perpendicular to GB) highlighted in red.

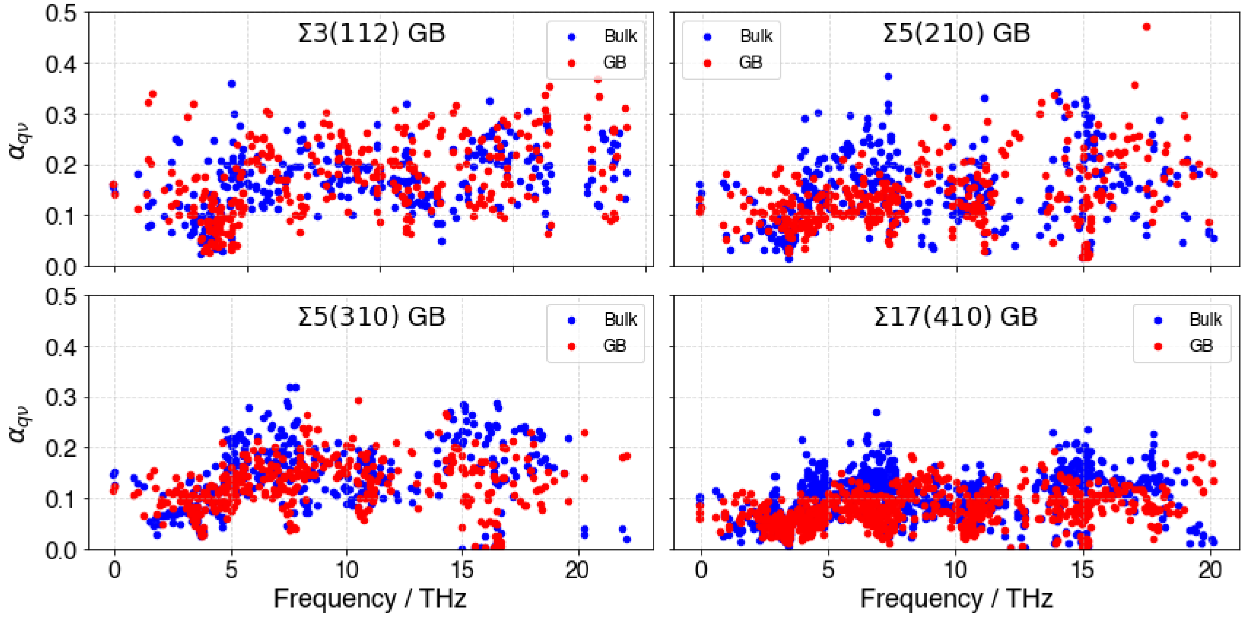

Figure S3: Alignment ($\alpha_{q\nu}$) of the eigenvectors of vibrations and the assumed Li-ion migration pathways (vacancy driven) for the Li ions in both the ϕ_{bulk} and ϕ_{GB} regions.
